# I Forgot It

**Published:** 1856-12

**Authors:** 


### "I FORGOT IT."

It's no such thing. It's the biggest fib a man ever told to say, "I forgot it." It's a libel on memory. It's a misnomer. There are "*sets*" in lying, as well as in society. Away down yonder, about circle one hundred below par, where mean lies are current, a falsehood which gives you no chance of getting behind it—"I forgot it" is one of them—refuge is taken in the untangible. The chambers of the memory are impenetrable. The true interpretation of "I forgot it" is, "*I don't care.*" This simple idea well merits reflection; its bearings are far reaching. "*I forgot it,*" is nothing more nor less than *indifference*, and *indifference* is uncourteous and dishonorable, wherever it is applied, in reference to an engagement or a duty. Does an honorable merchant "*forget*" an engagement? Can we imagine such a thing as a Christian "*forgetting*" his duty or a mother her first-born? Only the milk-and-water folk, the do-nothing-no-bodies can "forget,"—such as haven't "character" enough to keep them from falling to pieces.

